# An innovative model for predicting coronary heart disease using triglyceride-glucose index: a machine learning-based cohort study

**DOI:** 10.1186/s12933-023-01939-9

**Published:** 2023-08-04

**Authors:** Seyed Reza Mirjalili, Sepideh Soltani, Zahra Heidari Meybodi, Pedro Marques-Vidal, Alexander Kraemer, Mohammadtaghi Sarebanhassanabadi

**Affiliations:** 1 Yazd Cardiovascular Research Center, Non-Communicable Diseases Research Institute, Shahid Sadoughi University of Medical Sciences, Yazd, Iran; 2 Department of Internal Medicine, BH10-642, Rue du Bugnon 46, CH-1011 Lausanne, Switzerland; 3 Department of Health Sciences, Bielefeld University, Bielefeld, Germany

**Keywords:** TyG-index, Coronary heart disease, Machine learning, Cohort study, Predictive model

## Abstract

**Background:**

Various predictive models have been developed for predicting the incidence of coronary heart disease (CHD), but none of them has had optimal predictive value. Although these models consider diabetes as an important CHD risk factor, they do not consider insulin resistance or triglyceride (TG). The unsatisfactory performance of these prediction models may be attributed to the ignoring of these factors despite their proven effects on CHD. We decided to modify standard CHD predictive models through machine learning to determine whether the triglyceride-glucose index (TyG-index, a logarithmized combination of fasting blood sugar (FBS) and TG that demonstrates insulin resistance) functions better than diabetes as a CHD predictor.

**Methods:**

Two-thousand participants of a community-based Iranian population, aged 20–74 years, were investigated with a mean follow-up of 9.9 years (range: 7.6–12.2). The association between the TyG-index and CHD was investigated using multivariate Cox proportional hazard models. By selecting common components of previously validated CHD risk scores, we developed machine learning models for predicting CHD. The TyG-index was substituted for diabetes in CHD prediction models. All components of machine learning models were explained in terms of how they affect CHD prediction. CHD-predicting TyG-index cut-off points were calculated.

**Results:**

The incidence of CHD was 14.5%. Compared to the lowest quartile of the TyG-index, the fourth quartile had a fully adjusted hazard ratio of 2.32 (confidence interval [CI] 1.16–4.68, p-trend 0.04). A TyG-index > 8.42 had the highest negative predictive value for CHD. The TyG-index-based support vector machine (SVM) performed significantly better than diabetes-based SVM for predicting CHD. The TyG-index was not only more important than diabetes in predicting CHD; it was the most important factor after age in machine learning models.

**Conclusion:**

We recommend using the TyG-index in clinical practice and predictive models to identify individuals at risk of developing CHD and to aid in its prevention**.**

**Supplementary Information:**

The online version contains supplementary material available at 10.1186/s12933-023-01939-9.

## Introduction

CHD is a major public health challenge and contributes to the global disease burden. Despite improved prevention methods and treatment techniques [1, 2], it is still the leading cause of morbidity and mortality worldwide, representing 32% of all deaths [3], and an enormous stress on the national health finances [4, 5]. Thus, CHD risk assessment is a global public health priority.

Various CHD predictive models such as Framingham [6], Systematic COronary Risk Evaluation (SCORE) [7], Reynolds [8], American College of Cardiology/American Heart Association (ACC/AHA) [9], Joint British Societies’ consensus recommendations for the prevention of cardiovascular disease (JBS3) [10], Multi-Ethnic Study of Atherosclerosis (MESA) [11], QRISK [12] and prediction for atherosclerotic cardiovascular risk in China (China-PAR) [13], have been developed for predicting CHD incidence, but none has optimal predictive value [14]. All such models consider diabetes as an important CHD risk factor, but not one considers either insulin resistance or TG [14–17].

A better prediction of CHD may be possible by considering insulin resistance, which occurs years or even decades before diabetes [18]. Previous Mendelian randomized analyses, systematic reviews, and meta-analyses have advocated the association between insulin resistance and CHD by altering vascular wall responses for insulin and promoting atherosclerosis [19–21]. The hyperinsulinemic-euglycemic clamp test is the gold standard of insulin resistance measurement, but it is not applicable in clinical studies because of its invasive, complicated, and expensive protocol [22, 23]. Another validated index is the homeostasis model assessment of insulin resistance (HOMA-IR) calculated by dividing serum glucose by insulin concentrations. Circulating insulin concentration is not routinely measured in primary care. Moreover, it has limited value in subjects receiving subcutaneous insulin. Therefore, HOMA-IR is not a suitable index for primary prevention strategies [23]. The TyG-index is a logarithmized product of FBS and TG. It has been shown to correlate highly with the hyperinsulinemic-euglycemic clamp and HOMA-IR [24]. Moreover, it is a simple, low-cost protocol that can be used in all subjects regardless of their insulin treatment status [23]. Additionally, it contains TG, another risk factor for CHD [25, 26] as indicated by several studies; nonetheless, it has not been considered in previous models [6–13]. Therefore, it seems sensible to modify these models with the TyG-index and then evaluate their effectiveness.

Machine learning algorithms have been demonstrated to be extremely useful in predicting cardiovascular disease [27]. Their ability to capture complex interactions and nonlinear relationships between variables and outcomes makes them superior to standard statistical models [28]. Several studies have shown that machine learning algorithms outperform traditional models [29–31]. Despite this, no study has explored the impact of TyG-index on the prediction of CHD through machine learning. For these reasons, machine learning models should be chosen to fully assess how TyG-index and diabetes impact and interact with other variables when predicting CHD.

In view of the above, the primary objective of the current study was to investigate the association between the TyG-index and CHD in a 10-year prospective cohort study. The ultimate objective was to modify standard CHD predictive models through machine learning to determine whether the TyG-index functions better than diabetes as a CHD predictor.

## Methods

### Study population

This cohort study was conducted using data from Yazd Healthy Heart Project (YHHP) a population-based epidemiological study evaluating cardiovascular diseases and metabolic disorders [32].

In YHHP, 100 clusters and 20 families from each cluster were defined, and one adult (aged 20–74 years) from each family was randomly selected for participation and evaluation in the first phase conducted in 2005–2006 (n = 2000, men = 1000, women = 1000) [32].

After 10 years of follow-up (2015–2016), the participants were re-invited to Yazd Cardiovascular Research Centre (YCRC) to be re-evaluated [32].

### Included participants

**Fig. 1:**
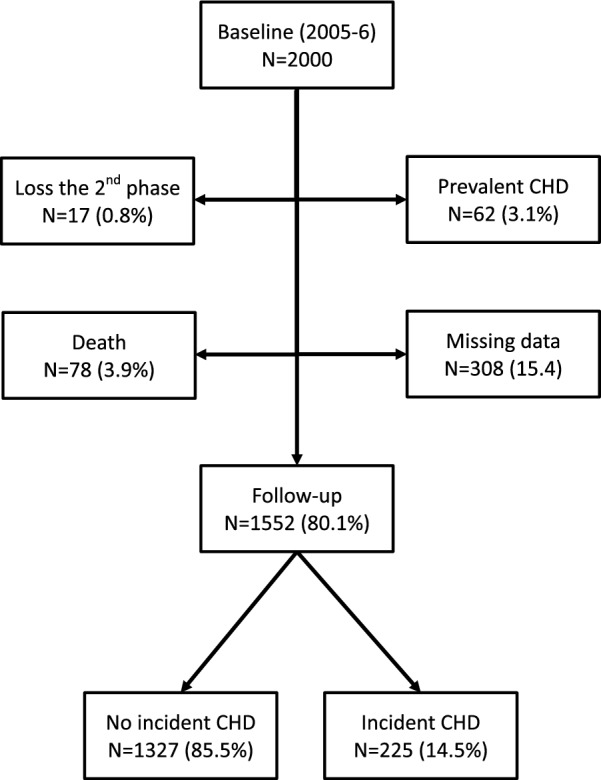
Flow diagram of participants attending the 10-year follow-up study

From the 2000 participants, 17 were omitted from the study due to loss during the second phase; from the 1983 individuals participating in the baseline examination, 62 were excluded due to diagnosis of CHD at baseline, 78 due to death during the study, and 308 due to missing data. The remaining 1552 participants (804 men, mean age 48.6 ± 14.7 years) were included in the present study (Fig. 1).

### Ethical approval

The present study was approved by the Shahid Sadoughi University of Medical Sciences ethics committee (ethics code: IR.SSU.REC.1401.069) and conducted based on the Declaration of Helsinki on medical research [33]. Informed consent was obtained from study participants during the initial and follow-up phases. The present research is reported based on strengthening the reporting of observational studies in the epidemiology (STROBE) statement [34].

### Biochemical analysis

Blood samples for laboratory tests were collected after overnight fasting. After centrifugation, serum uric acid (SUA), glucose, and TG were assessed using Pars Azmoon kits (Pars Azmoon Inc., Tehran, Iran). Bionic kits (Bionic Company, Tehran, Iran) were used to analyse lipid profiles (total cholesterol, low-density lipoprotein [LDL] and high-density lipoprotein [HDL]). All analyses were conducted using a biochemical auto-analyser (BT 3000, Italy) [32].

### Anthropometric and blood pressure measurements

Height was measured in both phases using a stadiometer fixed on a wall with no dents or bumps. While the participants were standing barefoot, their heels, hips, shoulders, and head touching the wall, and their head fixed horizontally to the nearest 0.5 cm. Participants were weighed to the nearest 0.1 kg in the first phase using a digital scale (Seca, Germany) with minimal clothing and in the second phase using another digital scale (Model BF511, Omron Co. Karada body scan, Osaka, Japan). The superior border of the iliac crest and widest part of the buttock were considered to measure waist and hip circumferences, respectively, to the nearest 0.1 cm using a non-stretchable tape.

An automatic digital blood pressure monitor (Omron, M6 comfort, Osaka, Japan) was used to measure blood pressure of the participants’ right arms, while they were in the sitting position. Blood pressure measurements were taken by a trained nurse twice, with an interval of 5 min [32].

### Data collection

Data including demographic features, education, physical activity, smoking habits, family history of premature CHD, and dietary habits were collected by completing questionnaires.

Trained interviewers completed questionnaires to assess physical activity, educational attainment, dietary habits and smoking status, in the first phase of the study. For educational attainment, participants were categorized as having a primary, high school, or academic education. Physical activity was assessed using the International Physical Activity Questionnaire (IPAQ) [35]. Participants were categorized as having low, moderate, or vigorous level of activity if their activity was < 600, 600–1200, or  > 1200 kilocalories/week, respectively. Participants were divided into groups of smokers or non-smokers based on their current smoking status. CHD occurrence in either father or brother less than 45 years of age, or mother or sister less than 55 years of age was defined as a family history of premature CHD [32]. A questionnaire was used to determine the use of fried foods, salt, removing poultry skin, eating out, meat consumption, and removing fat from meat.

CHD events were defined as occurrences of fatal or non-fatal CHD, myocardial infarction (MI), percutaneous coronary intervention (PCI), coronary artery bypass grafting (CABG), and new angina. The diagnosis of new angina was based on positive findings from the Rose angina questionnaire [36] in addition to positive electrocardiogram changes, elevated cardiac enzymes, and positive exercise tolerance test or coronary artery angiogram.

The time of outcome for fatal or non-fatal CHD, MI, CABG, positive exercise test, positive cardiac enzymes, and PCI was determined based on medical records. All Rose angina questionnaires [36] and electrocardiograms were investigated by an expert medical practitioner.

### Statistical analysis

Statistical analyses were performed with SPSS version 24.0 (IBM Corp., Armonk, NY, USA), Python 3, and R version 4.2.2 (www.R-project.org). Continuous variables were described as mean ± standard deviation (SD) and compared by independent T-test or ANOVA. Categorical variables were described as numbers (percentage) and compared using chi-square tests.

The TyG-index, the primary exposure variable of interest, was defined as:

$$TyG-index=ln\left(\frac{Tg\left(mg/dL\right)\times fasting\,glucose\left(mg/dL\right)}{2}\right)$$

and analysed as quartiles based on sex-specific distributions and as continuous measures. Multivariable Cox proportional hazard models were used to estimate the risk of CHD development. Four models were evaluated: model I was adjusted for age and sex; model II was further adjusted for physical activity, education, family history of premature CHD, and smoking; model III was further adjusted for total cholesterol, HDL, body mass index (BMI), waist-to-hip ratio, blood pressure, SUA, and LDL; and, model IV was further adjusted for consuming fried foods, adding salt, removing poultry skin, using high fat dairy products, dining out, meat consumption, and removing fat from meat. Finally, medication use was adjusted in our models for investigating whether it could modify the association.

The “OptimalCutpoints” [37] R package was used to assess TyG-index cut-off points that can predict CHD. We stratified these cut-points based on sex and diabetes status.

In accordance with previous studies [31, 38], we selected several machine-learning models to construct CHD-prediction models (logistic regression, decision tree, random forest, K nearest neighbor (KNN), and SVM). To simulate previous standard CHD predictor models, we investigated the literature and selected the common components between Framingham risk scores [6], SCORE CVD death risk score [7], QRISK risk calculator [12], Reynolds CVD risk score [8], ACC/AHA pooled cohort hard CVD risk calculator [9], JBS3 risk score [10], MESA risk score [11], and China-PAR risk predictor [13]. As a result of these investigations, age, sex, blood pressure, total cholesterol, HDL, waist-to-hip ratio, diabetes, smoking status, and family history of premature heart disease were considered in simulating a standard CHD prediction model. As part of the preprocessing of data, all missing values and evaluated outliers and highly correlated features were excluded. Because of imbalanced outcome data (14.5% incidence), we used SMOTE (over-sampling method) [39], which has been proven reliable for CHD [38]. After standardizing continuous variables and randomly splitting data into 70/30, we trained models on the larger part of the dataset and evaluated their performance on the smaller part. Afterward, we modified our dataset, by substituting the TyG-index for diabetes, and repeated the previous steps. For demonstrating the comparison of true positive, true negative, false negative, and false positive values of models, we used confusion matrices. We chose to use different color spectra to help illustrate the comparison, and make it easier to understand. To report model performance we calculated area under the curves (AUC), sensitivity, specificity, Cohen-kappa score Matthew's correlation coefficient, and F1-score. We used the generally accepted AUC index [31] and DeLong test [40] to compare the performance of these models. In order to make sense of machine learning models and counter the black box character of machine learning models, we used the “Dalex” library [41] to determine how much the performance of a model changes when a selected explanatory variable is removed.

## Results

Additional file 1: Table S1 summarizes the baseline characteristics of the study participants according to the follow-up process. Participants lost to follow-up were significantly older and less frequently male than participants who completed the follow-up.

Additional file 1: Table S2 explains the baseline characteristics of the study participants based on their gender.

The baseline characteristics of participants according to TyG-index quartiles are presented in Table 1. Participants in the highest quartile of serum TyG-index levels (TyG-index > 9.32) were older and had higher total cholesterol, TG, SUA, and fasting blood glucose levels, higher diabetes rates, blood pressure and anthropometric indices, lower HDL levels, and less education.

**Table 1:** Baseline clinical characteristics and biological variables of the participants according to serum TyG-index^a^ quartiles

	First	Second	Third	Fourth	p-value
Number of participants	358	386	386	392	
Age (years)	41.2 ± 15.7	48.2 ± 14.7	50.2 ± 13.6	53.8 ± 11.7	< 0.001
Mean follow-up (years)	9.9 ± 1.0	9.8 ± 1.0	9.9 ± 1.0	9.9 ± 1.3	0.29
Male (%)	171 (47.8)	219 (56.7)	195 (50.5)	200 (51)	0.09
Education (%)					< 0.001
Primary	159 (47.2)	211 (55.8)	238 (62.6)	277 (71.8)	
High school	144 (42.7)	116 (30.7)	102 (26.9)	87 (22.5)	
Academic	34 (10.1)	51 (13.5)	40 (10.5)	22 (5.7)	
Anthropometry					
Weight (Kg)	65.4 ± 12.4	71.1 ± 11.9	73.5 ± 12.8	74.7 ± 12.5	< 0.001
Weight/hip ratio	0.8 ± 0.1	0.9 ± 0.1	0.9 ± 0.1	0.95 ± 0.1	< 0.001
Waist circumference (cm)	85.9 ± 12.2	92.6 ± 10.8	96.9 ± 10.9	99.0 ± 10.5	< 0.001
BMI^b^ (Kg/m^2^)	23.9 ± 4.3	25.8 ± 4.1	27.2 ± 4.2	27.4 ± 4.0	< 0.001
Current smokers (%)	55 (15.4)	79 (20.5)	71 (18.4)	69 (17.6)	0.34
Physical activity (%)					0.01
Low	142 (61.5)	163 (63.2)	187 (70.3)	209 (74.6)	
Moderate	78 (33.7)	81 (31.4)	69 (25.9)	56 (20.0)	
Vigorous	11 (4.8)	14 (5.4)	10 (3.8)	15 (5.4)	
Blood pressure (mm Hg)					
Systolic	120.9 ± 13.9	127.4 ± 14.8	129.7 ± 14.6	134.1 ± 15.8	< 0.001
Diastolic	79.2 ± 7.9	82.1 ± 8.2	83.9 ± 9.1	84.9 ± 8.6	< 0.001
Diabetes (%)	5 (1.4)	20 (5.2)	43 (11.1)	187 (47.7)	< 0.001
Family history of CHD^c^	39 (11.0)	48 (12.6)	68 (18.2)	65 (16.8)	0.02
Blood levels (mg/dL)					
FBS^d^	81.4 ± 11.3	87.8 ± 14.9	96.2 ± 25.7	145.2 ± 69.6	< 0.001
Total cholesterol	172 ± 38.9	192.3 ± 39.2	207.1 ± 37.4	223.8 ± 47.8	< 0.001
LDL^e^	95.3 ± 33.1	110.0 ± 35.4	116.4 ± 33.0	114.6 ± 42.1	< 0.001
TG^f^	77.5 ± 19.6	128.5 ± 23.5	187.9 ± 40.9	305.7 ± 127.6	< 0.001
HDL^g^	58.0 ± 14.6	55.7 ± 12.7	52.2 ± 12.6	50.8 ± 14.2	< 0.001
SUA^h^	4.0 ± 1.1	4.4 ± 1.2	4.6 ± 1.3	4.6 ± 1.3	< 0.001

^a^Triglyceride-glucose index

^b^Body mass index

^c^Coronary heart disease

^d^Fasting blood sugar

^e^Low-density lipoprotein

^f^Triglyceride

^g^High-density lipoprotein

^h^Serum uric acid

### TyG-index and incidence of CHD

The overall incidence of new-onset CHD in the second visit was 14.5%. The incidence of CHD was 6.4%, 11.1%, 14%, and 26% in quartiles 1 to 4, respectively.

Compared with the Q1 group, the hazard ratio (HR) and 95% CI of CHD incidence in model I were 1.51 (0.91–2.51), 1.68 (1.03–2.74), and 2.63 (1.67–4.15) in Q2, Q3, and Q4 groups, respectively. After final adjustment (model IV), HR in Q4 was slightly decreased but still significant. Adjusted HR levels per 1-unit increase in TyG-index were 1.87 (1.59–2.21), 1.70 (1.35–2.14) and 2.16 (1.69–2.77) in the total sample, in men, and in women, respectively (Table 2).

**Table 2:** Risk of CHD^a^ according to quartiles of TyG-index^b^, overall and stratified by gender

	First	Second	Third	Fourth	P for trend
All participants					
Crude	1	1.94 (1.17–3.23)	2.20 (1.35–3.59)	4.04 (2.57–6.36)	< 0.001
Model I	1	1.51 (0.91–2.51)	1.68 (1.03–2.74)	2.63 (1.67–4.15)	< 0.001
Model II	1	1.79 (0.99–3.23)	1.71 (0.95–3.10)	2.92 (1.70–5.04)	< 0.001
Model III	1	1.76 (0.95–3.25)	1.65 (0.89–3.09)	2.45 (1.29–4.66)	0.007
Model IV	1	1.86 (0.96–3.61)	1.72 (0.87–3.41)	2.32 (1.16–4.68)	0.04
Non-Diabetic					
Crude	1	1.95 (1.16–3.28)	1.99 (1.18–3.34)	3.18 (1.90–5.34)	< 0.001
Model I	1	1.58 (0.94–2.66)	1.60 (0.95–2.69)	2.39 (1.42–4.02)	0.001
Model II	1	1.86 (1.00–3.43)	1.61 (0.85–3.06)	2.67 (1.44–4.93)	0.003
Model III	1	1.89 (0.99–3.6)	1.48 (0.73–2.97)	2.19 (0.98–4.91)	0.18
Model IV	1	1.89 (0.94–3.81)	1.54 (0.72–3.29)	2.07 (0.84–5.12)	0.14
Diabetic					
Crude	1	1.26 (0.64–2.46)	1.06 (0.53–2.13)	1.29 (0.67–2.48)	0.27
Model I	1	1.28 (0.65–2.51)	1.15 (0.57–2.31)	1.45 (0.75–2.82)	0.12
Model II	1	1.33 (0.62–2.86)	1.2 (0.54–2.69)	1.71 (0.78–3.75)	0.07
Model III	1	0.82 (0.34–1.99)	0.78 (0.30–2.03)	0.74 (0.26–2.14)	0.6
Model IV	1	0.63 (0.24–1.66)	0.56 (0.19–1.67)	0.45 (0.13–1.53)	0.1
Men					
Crude	1	1.70 (0.93–3.10)	1.64 (0.89–3.01)	3.06 (1.75–5.33)	< 0.001
Model I	1	1.59 (0.87–2.92)	1.56 (0.85–2.86)	2.45 (1.40–4.28)	0.001
Model II	1	1.96 (0.99–3.88)	1.69 (0.83–3.45)	2.84 (1.49–5.41)	0.001
Model III	1	2.02 (0.99–4.12)	1.45 (0.68–3.09)	1.88 (0.90–3.91)	0.02
Model IV	1	2.35 (1.06–5.20)	1.31 (0.57–3.04)	1.30 (0.56–3.01)	0.11
Women					
Crude	1	2.13 (0.84–5.41)	3.42 (1.47–7.93)	6.23 (2.81–13.8)	< 0.001
Model I	1	1.37 (0.53–3.51)	1.91 (0.81–4.49)	3.02 (1.34–6.82)	< 0.001
Model II	1	1.38 (0.41–4.68)	1.66 (0.54–5.10)	3.05 (1.04–8.93)	0.001
Model III	1	1.28 (0.35–4.60)	2.01 (0.61–6.56)	4.44 (1.26–15.7)	0.001
Model IV	1	1.20 (0.32–4.54)	2.14 (0.64–7.20)	4.65 (1.34–16.1)	0.004

Results are expressed as hazard ratio and (95% CI^c^). Model I: adjusted for age and sex; Model II: Adjusted for age, sex, smoking, physical activity, education, and family history; Model III: model II plus SUA^d^, HDL^e^, total cholesterol, BMI^f^, Waist to hip ratio, SBP^g^, DBP^h^, LDL^i^; Model IV: model III plus using fried food, adding salt, removing poultry skin, using high fat dairy products, dining out, meat consumption and removing its fat

^a^Coronary heart disease

^b^Triglyceride-glucose index

^c^Confidence interval

^d^Serum uric acid

^e^High-density lipoprotein

^f^Body mass index

^g^Systolic blood pressure

^h^Diastolic blood pressure

^i^Low density lipoprotein

When stratifying for gender, the association between TyG-index and risk of CHD in men was no longer significant after adjusting for laboratory markers and dietary patterns, yet it was still significantly associated with CHD in women: HR 4.65 (1.34–16.1) for Q4 compared to Q1. Diabetes medications confounded the association between TyG-index and CHD but dyslipidaemia treatment did not. A TyG-index higher than 9.07 in women and 8.92 in men had the highest sensitivity and specificity simultaneously for predicting CHD (Table 3).

**Table 3:** TyG-index^a^ cut-off points

Cut-Off-points	Maximum sensitivity and specificity simultaneously	Youden	Negative diagnostic ratio value	Positive diagnostic ratio value
Men	8.92	9.19	8.36	9.54
Women	9.07	8.94	9.04	9.10
Diabetic	9.73	9.46	8.79	10.67
Non-Diabetic	8.81	9.12	8.33	9.40
Total population	8.99	9.12	8.42	9.28

^a^Triglyceride-glucose index

^*^Positive diagnostic ratio; a particular value for the Positive Diagnostic Likelihood Ratio

^*^Negative diagnostic ratio value; a particular value for the Negative Diagnostic Likelihood Ratio

**Fig. 2:**
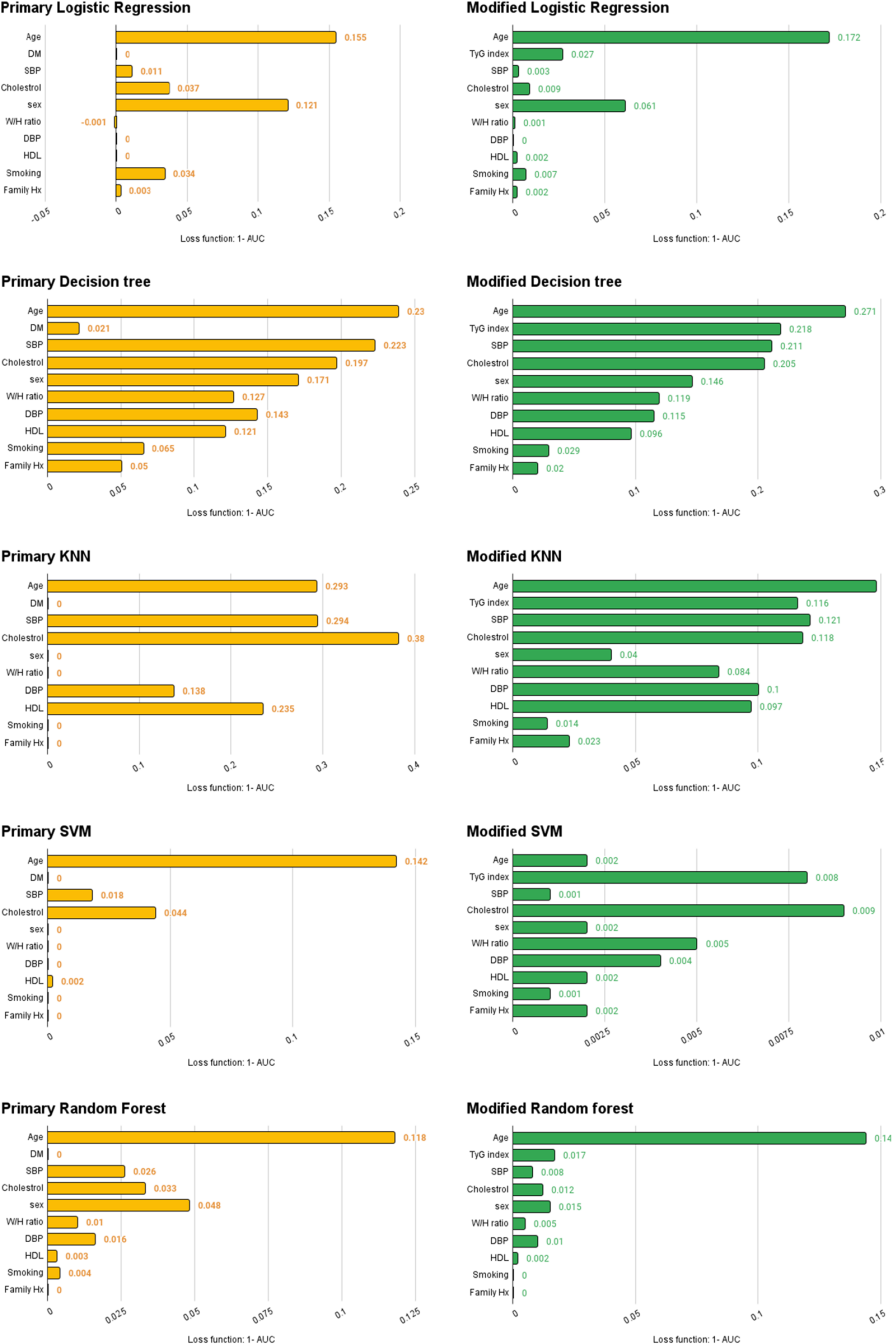
Impact of different components of machine learning models on predicting CHD

Table 4 shows the statistical functions, as well as the confusion matrices for predicting models consisting of true positive, false positive, true negative, and false negative values. Random forest models had the highest sensitivity and specificity. A significant improvement was seen in the SVM model after modification with the TyG-index. Other models showed no significant changes. In Fig. 2, all the components of these models are compared in terms of their impact on prediction**.** Eliminating diabetes decreased AUC by around 2% in the decision tree, whereas in other models, it did not affect AUC. Depending on the model, TyG-index removal decreased AUC from 1 to 22%. The current study showed that the TyG-index was much better than diabetes in predicting CHD; overall, it was the second most important factor after age.

**Table 4:**
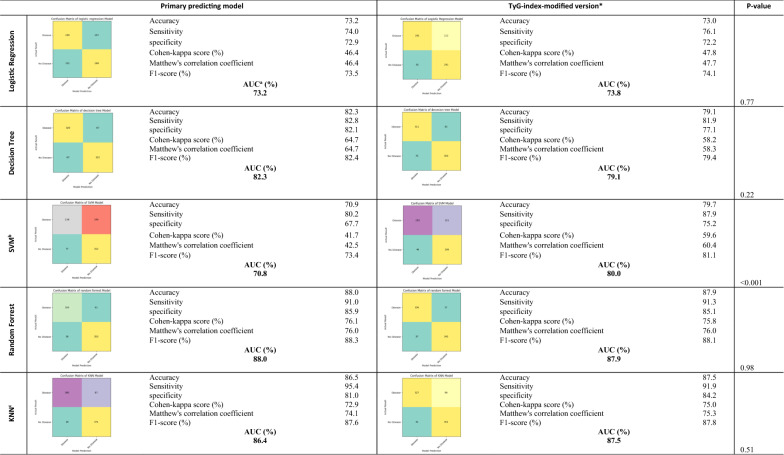
Comparison of the primary and TyG-index^a^-modified* versions of CHD^b^ predictive models using machine learning

^a^Triglyceride-glucose index

^b^Coronary heart disease

^c^High-density lipoprotein

^d^Area under the curve

^e^Support vector machine

^f^K nearest neighbor

^*^primary models were designed based on age, sex, blood pressure, smoking status, total cholesterol, HDL^c^, waist/hip ratio, family history of CHD and diabetes. In modified models diabetes was replaced by TyG-index

## Discussion

The results of this prospective cohort study in a community-based Iranian population followed for 9.9 years indicate that higher a TyG-index is associated with a higher risk of CHD. This association was more evident in females. Additionally, TyG-index outperformed diabetes in CHD prediction models.

### CHD and TyG-index association

An association between the TyG-index and CHD was previously confirmed in both observational [23, 42–49] and meta-analyses studies [19, 50, 51], but the inconsistency in predictive values, the incompleteness of confounding factors (especially diet and medications), and the need to investigate the association in non-diabetic patients in observational studies and heterogeneity in meta-analyses prompted the current study [19].

Previous studies have suggested TyG-index cutoff points of 9 and 9.323 for preventing CHD [52]. The results of the current study will aid healthcare providers in our region to screen their patients for a TyG-index of ≥ 8.42, which our results showed as having the highest negative predictive value, and to consider pharmacological treatment for values of ≥ 9.28, which had the highest positive predictive value in the current study, and to control those under 8.99, which had the highest sensitivity and specificity simultaneously.

### Mechanisms

FBS and TG are reflections of insulin resistance in the liver and adipocytes, respectively [53]. A combination of these two factors, the TyG-index demonstrated 96.5% sensitivity and 85% specificity for detecting insulin resistance, a better performance than that of HOMA-IR [51]. Resistance to insulin can trigger inflammatory processes, lipid metabolism deregulations, sympathetic nervous system over-activation, endothelial dysfunction, and eventually, thrombosis and CHD [43, 45, 46, 51, 54–57]. Therefore, the TyG-index can serve as a simple, practical, cost-effective, reproducible, and reliable surrogate marker for insulin resistance measurement in CHD prevention plans [54].

### TyG-index and gender

Studies have shown that the TyG-index plays a significant role in CHD incidence in women [42, 43, 45, 46, 54, 58, 59]. Nonetheless, one study reported a greater role in men [60], and another found no differences between genders [55]. The current study found an association in both genders which persisted only in women after multivariable adjustment. This finding may be explained by the fact that nearly half of the female participants were over 50 years of age and susceptible to menopause at the baseline. Insulin resistance and higher CHD risks can occur after menopause because of decreasing estrogen levels [45, 46, 54, 55, 59]. Furthermore, the TyG-index was an independent risk factor for CHD until model II in non-diabetic participants. The lack of association in diabetic participants may have been due to lifestyle changes and medication consumption during the 10 years of follow-up [61]. Our analysis showed that diabetes treatment made the association non-significant. The first line of diabetes treatment is metformin which can decrease insulin resistance [62], confirming the insignificant association between the TyG-index and CHD in diabetic participants.

### Prediction of CHD based on TyG-index

Previous studies have suggested that the TyG-index predicts cardiovascular events more accurately than hemoglobin A_1_c [23]. In addition, several studies have implicated that adding the TyG-index to the Framingham risk score can increase its predictive power [48, 49] Previous studies concluded that SVM and random forest were the most effective model for predicting CHD [38, 63, 64], the current study found that random forest achieved the highest AUC. In both random forest and SVM models, diabetes played no role, while the TyG-index was the second most influential component. The current study found that the use of the TyG-index instead of diabetes in machine learning models can significantly improve the predictive power of CHD predicting models. Machine learning models demonstrated that the TyG-index was not only more important than diabetes in predicting CHD, but it also was the most important factor after age. To the best of our knowledge, the TyG-index is not used in any clinical guideline [19], but the American Diabetes Association (ADA) suggested in 2022 that patients with elevated TG levels (≥ 150 mg/dL [1.7 mmol/L]) should implement enhanced lifestyle interventions and optimal glycemic control [65]. Our findings advocate the inclusion of the TyG-index in future CHD prevention guidelines.

### Strengths and limitations

The following strengths of the current study should be noted. This study is the first to evaluate the predictive power of TyG-index in CHD using machine-learning techniques. To the best of our knowledge, the optimal cut-off points had not previously been evaluated in the Iranian population. The community-based prospective nature of our study and definite outcome determination minimize the chance of reverse causation and recall bias. Including both old and young populations was another advantage the current study had over others, as most previous studies recruited middle-aged and older adults. Furthermore, the current study attempted to ameliorate the adjustment of confounders by adding family history of premature CHD, medication use, dietary habits, complete lipid profile components and all anthropometric features to our models. The long follow-up time in the present study acts as a double-edged sword; indeed, it can reflect the lifetime risk of CHD, but on the other hand, our inability to evaluate and control voluntarily health check-ups or lifestyle changes during the ten-year study period may have affected our findings. Compared to previous studies, we had an identical method for defining of CHD by investigating ECGs, cardiac enzymes, using the Rose angina questionnaire, exercise tolerance test, and coronary artery angiogram.

This study had several limitations. First, it was embedded in an observational setting, and despite a wide range of adjustments, we cannot rule out the possibility of unmeasured confounders. Single baseline TyG-index investigation may incline our results to intra-individual variation. Second, we may have observed gender-specific results due to the lack of data on menopausal status. Third, only Iranian subjects were included, so our findings might not be generalizable to other countries.

## Conclusion

The TyG-index can be used in clinical practice and predictive models as a highly valuable index for predicting and preventing CHD, but further studies are needed to validate our findings.

### Supplementary Information

**Additional file 1: Table S1.** Baseline clinical characteristics and biological variables of the participants according to inclusion process. **Table S2.** Comparison of baseline clinical characteristics and biological variables between genders.

## Data Availability

The datasets used and/or analyzed during the current study are available from the corresponding author on reasonable request.

## Abbreviations

TyG-index: Triglyceride-glucose index
CHD: Coronary heart disease
CI: Confidence interval
HOMA-IR: Homeostatic Model Assessment for Insulin Resistance
YHHP: YAZD healthy heart project
TG: Triglyceride
LDL: Low-density lipoprotein
HDL: High-density lipoprotein
FBS: Fasting blood sugar
SUA: Serum uric acid
IPAQ: International Physical Activity Questionnaire
SD: Standard deviation
BMI: Body mass index
KNN: K nearest neighbour
SVM: Support vector machine
ADA: American Diabetes Association
ECG: Electro-cardio-gram
AUC: Area under the curves
SCORE: Systematic COronary Risk Evaluation
ACC/AHA: American College of Cardiology/American Heart Association
China-PAR: Prediction for atherosclerotic cardiovascular risk in China
MESA: Multi-Ethnic Study of Atherosclerosis
JBS3: Joint British Societies’ consensus recommendations for the prevention of cardiovascular disease
