# Supplementary material for: An innovative model for predicting coronary heart disease using triglyceride-glucose index: a machine learning-based cohort study
Source: Cardiovasc Diabetol. 2023 Aug 4;22:200. doi: 10.1186/s12933-023-01939-9 (PMC10403891; doi:10.1186/s12933-023-01939-9)
Supplement: Supplementary file 1 — Additional file 1: Table S1. Baseline clinical characteristics and biological variables of the participants according to inclusion process. Table S2. Comparison of baseline clinical characteristics and biological variables between genders. [file 12933_2023_1939_MOESM1_ESM.docx]

|  | **Included** | **Number of missing data in included group (%)** | **Excluded** | **Total** | **p-value** |
| --- | --- | --- | --- | --- | --- |
| **Number of participants** | 1552 | - | 448 | 2000 |  |
| **Age (years)** | 48.6 ± 14.7 | 0 | 49.1 ± 16.8 | 48.7±15.2 | <0.001 |
| **Male (%)** | 804 (51.8) | 0 | 193 (43.2) | 998 (49.9) | 0.01 |
| **Education (%)** |  | 41 (2.6) |  |  | 0.09 |
| Primary | 903 (59.8) |  | 283 (64.3) | 1186 (60.8) |  |
| High school | 460 (30.4) |  | 127 (28.9) | 587 (30.1) |  |
| Academic | 148 (9.8) |  | 30 (6.8) | 178 (9.1) |  |
| **Anthropometry** |  |  |  |  |  |
| Weight (Kg) | 71.3± 12.9 | 10 (0.6) | 68.9± 13 | 70.8±12.9 | 0.96 |
| Waist/hip ratio | 0.9±0.1 | 13 (0.8) | 0.9±0.1 | 0.9±0.1 | 0.73 |
| Waist circumference (cm) | 93.8±12.1 | 8 (0.5) | 92.4±12.6 | 93.5±12.2 | 0.64 |
| BMI^[[1]](#footnote-1)^ (Kg/m^2^) | 26.2±4.3 | 15 (1) | 26±4.9 | 26.1±4.5 | 0.64 |
| **Current smokers (%)** | 280 (18.1) | 5 (0.3) | 69 (15.6) | 349 (17.6) | 0.23 |
| **Physical activity (%)** |  | 493 (31.8) |  |  | 0.44 |
| Low | 719 (67.9) |  | 223 (71.7) | 942 (68.8) |  |
| Moderate | 290 (27.4) |  | 75 (24.1) | 365 (26.6) |  |
| Vigorous | 50 (4.7) |  | 13 (4.2) | 63 (4.6) |  |
| **Blood pressure (mm Hg)** |  |  |  |  |  |
| Systolic | 128.2±15.5 | 0 | 127.9±16.4 | 128.5±15.7 | 0.98 |
| Diastolic | 82.7±8.8 | 0 | 82±9.2 | 82.5±8.9 | 0.15 |
| **Diabetes (%)** | 264 (17) | 0 | 93 (20.9) | 357 (17.9) | 0.06 |
| **Blood levels (mg/dL)** |  |  |  |  |  |
| FBS^[[2]](#footnote-2)^ | 103.1±46 | 8 (0.5) | 102 ±44.2 | 102.8±45.6 | 0.92 |
| Total cholesterol | 199.3±45.1 | 7 (0.5) | 198.3±93.9 | 199.1±59.4 | 0.23 |
| LDL^[[3]](#footnote-3)^ | 109.2±36.8 | 51 (3.3) | 106.2±35.1 | 108.5±36.4 | 0.4 |
| TG^[[4]](#footnote-4)^ | 179.8±109.2 | 7 (0.5) | 170.7±103.1 | 175.4±107.9 | 0.29 |
| HDL^[[5]](#footnote-5)^ | 54.1±13.8 | 8 (0.5) | 53.2±13.2 | 53.9±16.7 | 0.41 |
| SUA^[[6]](#footnote-6)^ | 4.4±1.3 | 7 (0.5) | 4.7±6.7 | 4.5±3.3 | 0.06 |
| **TyG-index^[[7]](#footnote-7)^** | 8.9±0.7 | 8 (0.5) | 8.9±0.7 | 8.9±0.7 | 0.31 |

**Additional file Table 1.** Baseline clinical characteristics and biological
variables of the participants according to inclusion process

**Additional file Table 2.** Comparison of baseline clinical characteristics and
biological variables between genders

|  | **Male** | **Female** | **p-value** |
| --- | --- | --- | --- |
| **Number of participants** | 804 | 748 |  |
| **Age (years)** | 48.4±14.9 | 48.8±14.5 | 0.6 |
| **Mean follow-up (years)** | 9.8±1 | 9.9±1.1 | 0.3 |
| **Education (%)** |  |  | <0.001 |
| Primary | 377 (48.7) | 526 (71.4) |  |
| High school | 279 (36) | 181 (24.6) |  |
| Academic | 118 (15.2) | 30 (4.1) |  |
| **Anthropometry** |  |  |  |
| Weight (Kg) | 74.6±12.3 | 67.8±12.5 | <0.001 |
| Weight/hip ratio | 0.92±0.08 | 0.90±0.1 | <0.001 |
| Waist circumference (cm) | 93.5±11.8 | 94±12.4 | 0.4 |
| BMI^[[8]](#footnote-8)^ (Kg/m^2^) | 25.2±3.8 | 27.2±4.6 | <0.001 |
| **Current smokers (%)** | 272 (34) | 8 (1.1) | <0.001 |
| **Physical activity (%)** |  |  | 0.02 |
| Low | 436 (68.7) | 283 (66.7) |  |
| Moderate | 161 (25.4) | 129 (30.4) |  |
| Vigorous | 38 (6) | 12 (2.8) |  |
| **Blood pressure (mm Hg)** |  |  |  |
| Systolic | 129.5±14.8 | 126.9±16 | 0.001 |
| Diastolic | 83.4±8.7 | 81.8±8.7 | <0.001 |
| **Diabetes(%)** | 129 (16) | 135 (18) | 0.3 |
| **Family history of CAD(%)** | 97 (12.3) | 125 (16.7) | 0.01 |
| **Blood levels (mg/dL)** |  |  |  |
| FBS^[[9]](#footnote-9)^ | 101.2±40.5 | 105±51.1 | 0.1 |
| Total cholesterol | 192.4±41.9 | 206.7±47.3 | <0.001 |
| LDL^[[10]](#footnote-10)^ | 104.4±35.1 | 114.1±37.6 | <0.001 |
| TG^[[11]](#footnote-11)^* | 150(106-219) | 149(101-219) | 0.4 |
| HDL^[[12]](#footnote-12)^ | 51.9±13.1 | 56.5±14.1 | <0.001 |
| SUA^[[13]](#footnote-13)^ | 4.9±1.2 | 3.9±1 | <0.001 |

*Results are expressed as number of participants (percentage) for categorical variables and as average ±standard deviation for continuous variables Between-group comparisons
performed using chi-square for categorical variables and analysis of variance for continuous variables*TG are reported as median(interquartile ranges)*

1. Body mass index [↑](#footnote-ref-1)
2. Fasting blood sugar [↑](#footnote-ref-2)
3. Low-density lipoprotein [↑](#footnote-ref-3)
4. Triglyceride [↑](#footnote-ref-4)
5. High-density lipoprotein [↑](#footnote-ref-5)
6. Serum uric acid [↑](#footnote-ref-6)
7. Triglyceride-glucose index [↑](#footnote-ref-7)
8. Body mass index [↑](#footnote-ref-8)
9. Fasting blood sugar [↑](#footnote-ref-9)
10. Low-density lipoprotein [↑](#footnote-ref-10)
11. Triglyceride [↑](#footnote-ref-11)
12. High-density lipoprotein [↑](#footnote-ref-12)
13. Serum uric acid [↑](#footnote-ref-13)
